# Mental Simulation of Painful Situations Has an Impact on Posture and Psychophysiological Parameters

**DOI:** 10.3389/fpsyg.2017.02012

**Published:** 2017-11-21

**Authors:** Thierry Lelard, Olivier Godefroy, Said Ahmaidi, Pierre Krystkowiak, Harold Mouras

**Affiliations:** ^1^EA 3300, Adaptations Physiologiques à l'Exercice et Réadaptation à l'Effort, UFR des Sciences du Sport, Université de Picardie Jules Verne, Amiens, France; ^2^EA 4559, Laboratoire de Neurosciences Fonctionnelles et Pathologies, UFR de Médecine, Université de Picardie Jules Verne, Amiens, France; ^3^Structure Fédérative de Recherche en Santé CAP-Santé, Université de Picardie Jules Verne, Amiens and Université de Reims-Champagne-Ardennes, Reims, France; ^4^Service de Neurologie, CHU Amiens, Amiens, France; ^5^EA 7273, Centre de Recherche en Psychologie: Cognition, Psychisme et Organisations, UFR de Sciences Humaines Sciences Sociales et Philosophie, Département de Psychologie, Université de Picardie Jules Verne, Amiens, France

**Keywords:** embodiment theory, empathy for pain, posturography, embodiment, motor correlates

## Abstract

Embodiment is made possible by the ability to imagine ourselves in a particular situation (mental simulation). Postural changes have been demonstrated in response to painful situations, but the effect of an implicit instruction has not been studied. The present study was designed to record differential responses according to whether or not subjects were instructed to imagine themselves in a painful or non-painful situation. Painful stimuli and instructions to mentally simulate the displayed situation were hypothesized to induce postural changes that could be demonstrated by changes in the center of pressure (COP) trajectory compared to viewing the same stimuli with no instructions. We hypothesized that mental simulation of a painful situation would induce embodiment of the emotional situation as reflected by posterior displacement of the COP and physiological responses as compared to passive observation of the same visual scene. Thirty-one subjects participated in this study while standing quietly on a posturographic platform with presentation of visual stimuli depicting scenes defining three experimental conditions (painful, non-painful and neutral situations) for 12 s. Physiological measurements [heart rate (HR) and electrodermal activity] and postural responses (COP displacements) were recorded in response to the stimuli with or without instructions to imagine themselves in the situation. Time-course analyses (1 s sliding window) were conducted for several postural parameters, HR and electrodermal response. An interaction effect (instruction × stimuli × time) demonstrated that mental simulation induced posterior displacement of the mean position of the COP at different times during presentation of visual stimuli (4 s; 9–12 s). An effect of instruction was reported for HR (HR was higher in the mental simulation condition), while a stimulation effect was reported only for HR (lower for painful stimuli than for non-painful stimuli). The results of time-course analyses demonstrated embodiment of painful situations by postural control modulations and physiological changes depending on whether or not the participants were instructed to imagine themselves in the situation.

## Introduction

According to embodiment theories, experiencing emotional states affects motor systems (Michalak et al., 2009). Embodiment is made possible by the ability to imagine ourselves in a situation, also defined as mental simulation. Mental simulation is an important feature in terms of adaptive behavioral skills by allowing the subject to anticipate the result of a movement, an important process for understanding other people's behavior and contributing to facilitate interactions with the environment. Instruction to simulate another person's behavior or to imagine personally experiencing a visually displayed situation involves simulation processes and activation of internal models (Zahavi, 2008). Hétu et al. (2013) demonstrated that mental simulation of a movement recruits identical neural circuits in the frontoparietal areas as the real movement. Mental simulation of movements is therefore used in various domains, such as sports or music training or neurorehabilitation (Stins et al., 2015). Embodied simulation theory explains the mechanisms by which we can understand another person's actions and the induction of bodily expression of emotion. Mental simulation of motor or emotional situations also induces changes in the participant's physiological state. We have recently demonstrated (Lelard et al., 2013) that simulation of a painful scene induces postural and physiological changes compared to simulation of a non-painful scene. In this study, we demonstrated that bodily behavior is affected by the valence of the simulated action (painful vs. non-painful situation), as participants demonstrated a general freezing behavior while imagining themselves in a painful situation compared to a non-painful situation. This general freezing behavior corresponds to a complex and flexible defensive response (such as fleeing). Eilam (2005) considered that both of these defensive responses are probably controlled by different motor systems that are interconnected to allow fast switching between these behaviors, as required for an effective and versatile response (Eilam, 2005). Freezing reactions are characterized by reduced body sway and bradycardia (Volchan et al., 2017).

In previous studies, empathy for pain has been shown to be an efficient functional context to explore the effect of simulation of an emotional situation (Montalan et al., 2012; Bucchioni et al., 2015). Price (2000) considered that “the affective experience of pain signals induces an aversive state that motivates behavior to terminate, reduce, or induce escape to the exposure of the noxious stimulation.” According to the perception-action model (Preston and de Waal, 2002), empathy automatically activates somatic and autonomic responses and embodiment of a painful situation might therefore be an efficient functional context to study emotional information processing associated with the promotion of protective or recovery visceromotor and behavioral responses.

Posturography determines displacements of the Center of Pressure (COP) and is appropriate to demonstrate postural changes and quantify body movements, such as approach-withdrawal behavior (Gurfinkel, 1973; Winter et al., 1990). In recent studies, this method was used to record motor responses induced by emotional stimuli while subjects remained in bipedal and/or unipedal stance. Presentation of emotional pictures (International Affective Picture System, IAPS; Lang et al., 2008) has been shown to induce approach-withdrawal behavior (Hillman et al., 2004) or freezing responses (Hillman et al., 2004; Azevedo et al., 2005; Stins and Beek, 2007; Lelard et al., 2013). However, most of these studies assessed mean postural responses induced over periods of 6 to 50 s. Temporal displacements of the COP in response to emotional visual stimulation can be used to differentiate body responses according to the valence of the stimulus (Hagenaars et al., 2014a,b; Lelard et al., 2014). In these studies, temporal analyses of COP displacement were conducted with a 1-s window. The effect of emotional stimuli was reported in the mean position of the COP in the anteroposterior direction. Posterior displacement of the mean COP position was reported after 3 s in response to aversive stimuli compared to neutral stimuli (Lelard et al., 2014) and a reduction of sway path was reported after 1.5 s in response to unpleasant video images compared to pleasant video images (Hagenaars et al., 2014b). While differences between mental simulation of painful and non-painful situations demonstrated that the emotional valence of the situation modulates postural responses (Lelard et al., 2013), the effects of mental simulation were not tested, as, in the absence of a control condition, this study was unable to determine whether the reported effects were due to embodiment of the situation or to the valence of the visual scene. However, several recent studies have demonstrated that motor imaging of movements induced different postural control responses when the participants imagined themselves performing a movement or imagined another person performing the same movement (Rodrigues et al., 2010; Grangeon et al., 2011; Stins et al., 2015). We therefore hypothesized that the instruction to imagine ourselves in a situation would induce embodiment of the emotional situation and would consequently change the responses compared to simple observation of the same visual scene.

The aim of this study was to record differential postural and physiological responses according to whether or not subjects were instructed to imagine themselves in a painful or non-painful situation in the functional context of empathy for pain. Painful visual scenes and instructions to embody the displayed situation were hypothesized to induce postural and physiological changes that could be demonstrated by posterior displacement of the trajectory of the COP (avoidance strategy) and by bradycardia as compared to viewing the same stimuli with no instructions.

## Methods

### Participants

Thirty-one participants (14 males, 17 females; mean age and *SD* = 22.3 ± 3.7) with no known visual or motor impairment and no previous or current treatment for psychiatric or neurological disorders were included in this study. All participants signed an informed consent form. Experimental procedures were conducted in accordance with the ethical standards of the Declaration of Helsinki and were approved by the local ethics committee (CPP Nord Ouest 2, Amiens, France).

### Stimuli

Ten static visual stimuli depicting feet or hands in painful situations (five pictures) or non-painful situations were selected from a large database (Jackson et al., 2005) and were demonstrated to induce differential postural responses (Lelard et al., 2013). Presentation of stimuli was controlled by a computer running E-Prime software (Psychology Software Tools, Inc., Pittsburgh, PA, USA).

### Procedure

Participants stood barefoot in the middle of the posturographic platform and were asked to maintain a comfortable bipedal stance with their arms hanging relaxed alongside their body and their feet pointing 30° outwards. Visual stimuli were then presented in front of the participants using a video projector. A trigger corresponding to each type of emotional stimulus (Painful, Non-painful and Neutral stimuli) for each picture was sent to a Biopac MP150 system (BIOPAC Inc., Goleta, CA, USA).

**Figure 1:**
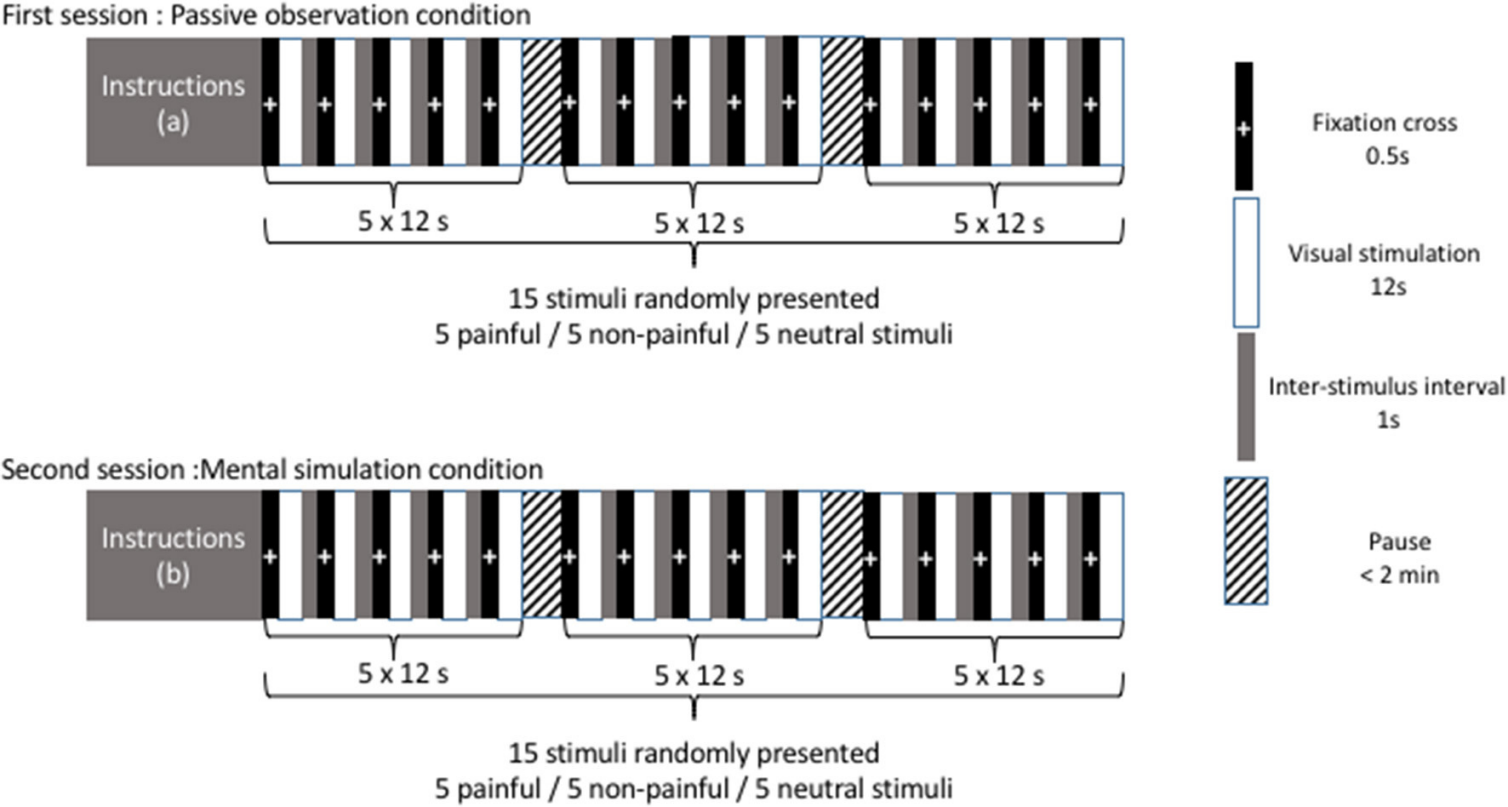
Presentation of pictures and timing. Instructions **(a)** maintain a comfortable bipedal stance and remain as motionless as possible without any voluntary movements. Instructions **(b)** maintain a comfortable bipedal stance and remain as motionless as possible without any voluntary movements. Imagine that you are personally experiencing the situations you are about to see.

Presentation of pictures and timing are illustrated in Figure 1. In a first recording session (corresponding to the “passive observation condition”), participants were instructed to watch the images presented without any additional instruction other than “remain as motionless as possible, do not make any voluntary movements.” During this first session, the 10 pictures (5 painful and 5 non-painful images) and 5 neutral stimuli (corresponding to a gray background) were randomly presented. The trial sequence started with a fixation cross for 0.5 s. The stimulus was then presented for a duration of 12 s and a 1-s inter-stimulus interval was added. After presentation of three stimuli, participants were invited to stretch their legs and sit comfortably to avoid tiredness. In a second session (corresponding to the “mental simulation condition”), participants were instructed to imagine that they had personally experienced the situations they were about to see. The same stimuli (5 painful, 5 non-painful, and 5 neutral stimuli) were presented randomly while the participants maintained the same comfortable bipedal stance with the instruction: “remain as motionless as possible, do not make any voluntary movements.”

### Assessment of posturographic and physiological data

Posturographic and physiological data were recorded at a rate of 1,000 Hz using a MP-150 Biopac System and simultaneously computed by AcqKnowledge software (Biopac Inc., Santa Barbara, CA). Displacements of the COP were recorded during the rest stance by a posturographic platform (Satel, Blagnac, France). Analog data from three strain gauges were recorded and anteroposterior (AP) and mediolateral (ML) displacements of the COP were computed by AcqKnowledge software (Biopac Inc., Santa Barbara, CA). Electrodermal activity (EDA) was recorded with two Ag/AgCl electrodes (GSR100C, Biopac Inc., Santa Barbara, CA) filled with isotonic paste attached to the volar surface of the index and middle fingers of the subject's hand. A constant-voltage device was used to deliver 0.5 V between electrodes. Electrocardiogram (ECG) signals were recorded with a standard Lead-II electrocardiogram using three disposable electrodes (EL503, BIOPAC Inc., Goleta, CA, USA).

### Data analysis

Data were extracted from AcqKnowledge software and all dependent variables were calculated using a custom Matlab function. The following indices were calculated for each trial: (i) COP position in the anteroposterior direction (COP-AP), reflecting the extent to which a participant leaned toward the anterior or posterior directions; (ii) heart rate (HR) was calculated from ECG data by AcqKnowledge software (HR); (iii) the EDA signal was integrated and expressed in arbitrary units. For each trial, the mean postural and physiological responses were calculated for a 1 s sliding window. The mean results for both painful and non-painful situations were determined for the 5 trials for each experimental condition. Time-course analysis was also performed (by averaging the over a 1 s sliding window) in order to determine the time of onset of any differences in COP-AP, HR, and EDA.

### Statistical analysis

Two-way (Instruction: mental simulation/passive observation), three-way (Stimuli: painful visual scene/non-painful visual scene/no visual scene) and 12-way [Time (s): 1–12 window] repeated measures ANOVA of postural and physiological data were performed to study temporal responses to instructions and visual stimuli. Between-condition differences were tested with a Bonferroni *post-hoc* test. The limit of statistical significance was defined as *p* < 0.05 for all analyses.

## Results

### Postural responses to visual stimuli

**Figure 2:**
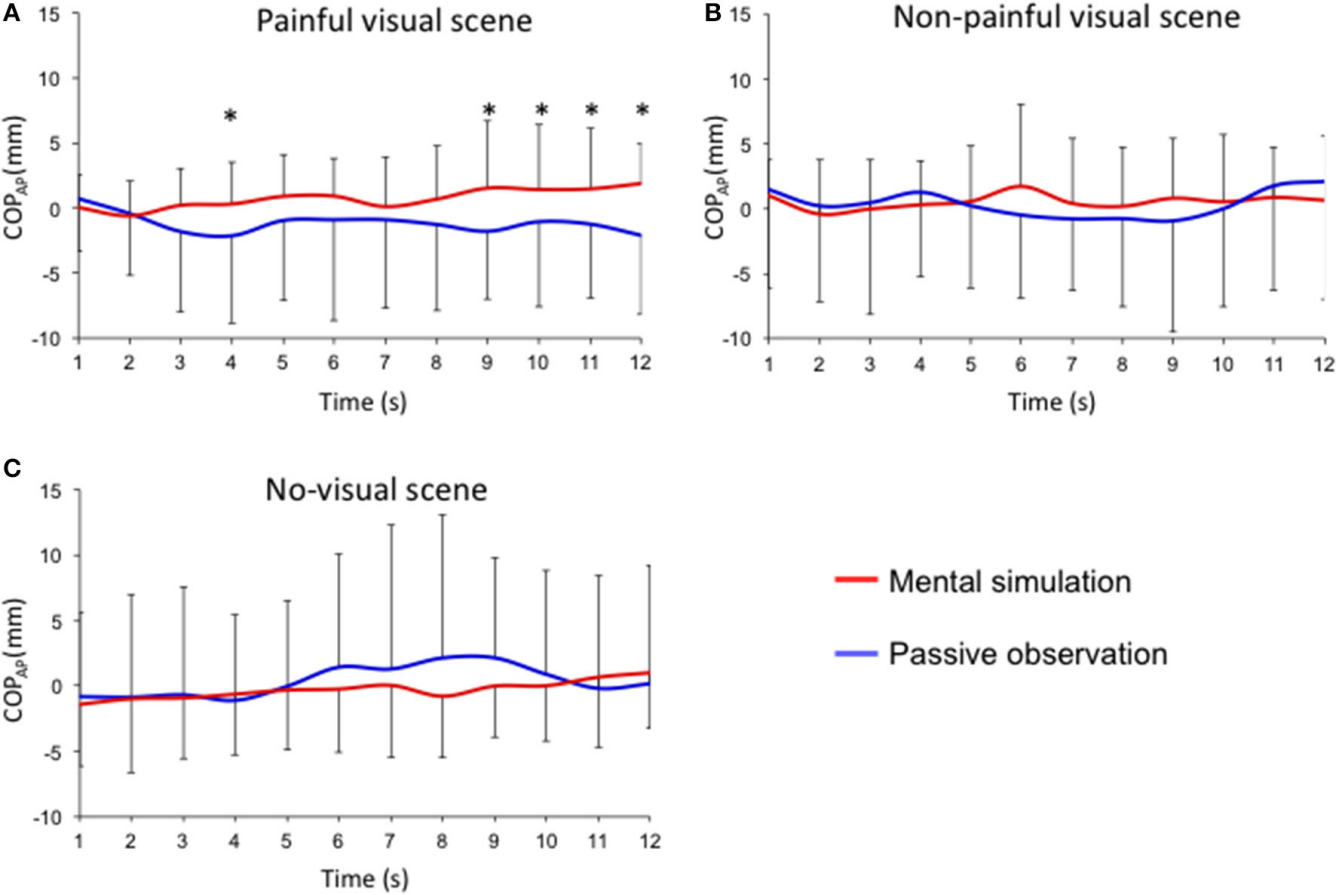
Time-course of the anteroposterior position of the COP_AP_ for each experimental condition (mental simulation vs. passive observation conditions). Mean sliding window (1 s) for the three experimental conditions defined by the presentation of **(A)** painful visual scene; **(B)** non-painful visual scene; **(C)** no visual scene. ^*^p < 0.05.

**Figure 3:**
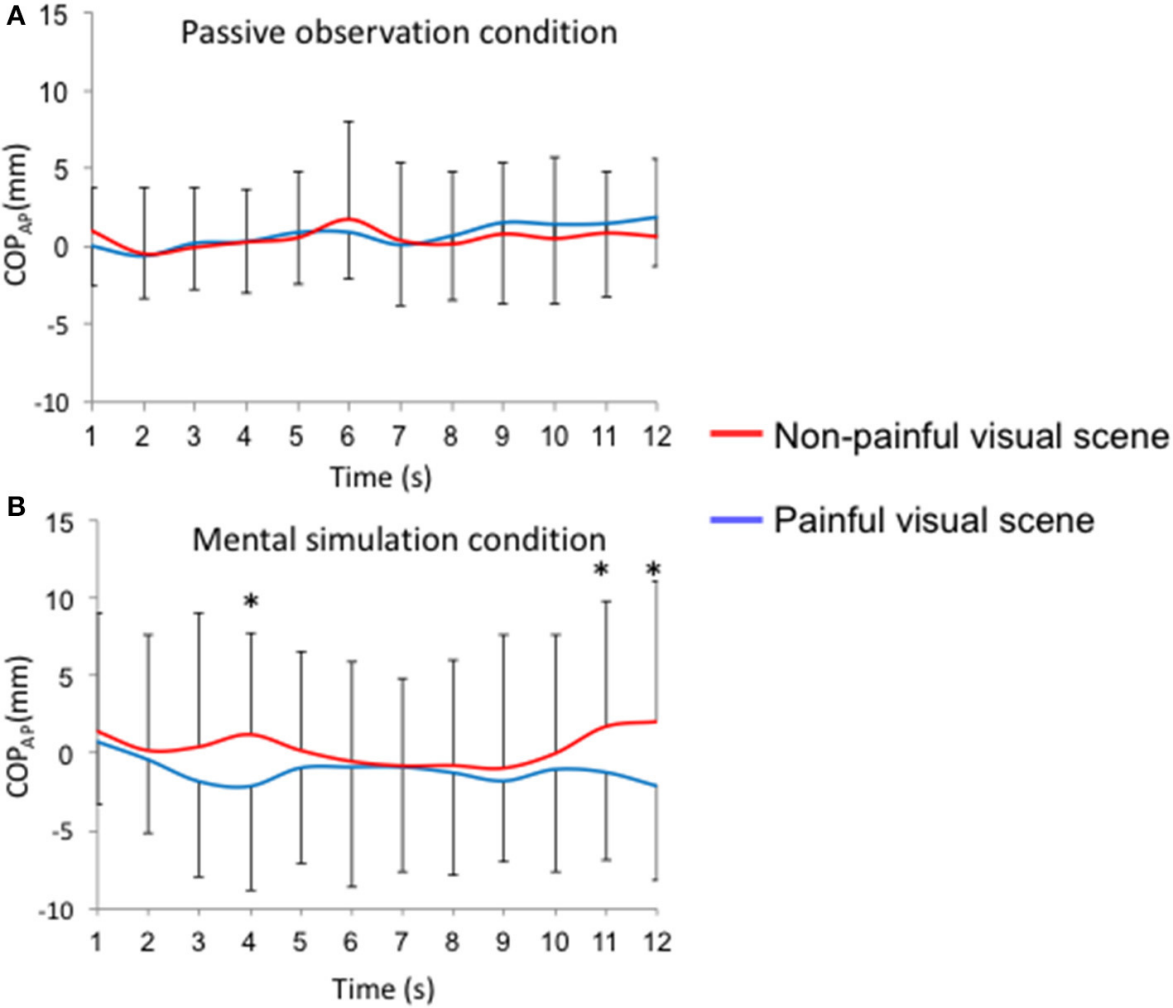
Time-course of the anteroposterior position of the COP_AP_ for each visual stimulus (painful vs. non-painful visual scenes). Mean sliding window (1 s): **(A)** passive observation condition; **(B)** mental simulation condition, ^*^p < 0.05.

Figures 2, 3 describe COP-AP during the 12-s presentation of painful and non-painful stimuli. An interaction effect (instruction × stimuli × time) was reported for the mean position of the COP in the anteroposterior direction (*F* = 2.013; *p* < 0.05; effect size: 0.07). *Post-hoc* tests showed that presentation of painful situations induces different postural responses at the 4th (*p* < 0.05), 9th (*p* < 0.05), 10th (*p* < 0.05), 11th (*p* < 0.05), and 12th seconds (*p* < 0.05) when the participants were asked to imagine themselves in the situation compared to simple passive observation (Figure 2A). Posterior displacement of COP-AP was observed in the mental simulation condition compared to passive observation for the 4th (−2.1 ± 6.8 mm vs. 0.3 ± 3.3 mm; *p* < 0.05), 9th (−1.8 ± 5.3 mm vs. 1.5 ± 5.2 mm; *p* < 0.05), 10th (−1.0 ± 6.6 mm vs. 1.39 ± 5.1 mm; *p* < 0.05), 11th (−1.2 ± 5.7 mm vs. 1.4 ± 4.7 mm; *p* < 0.01), and 12th seconds (−2.1 ± 6.1 mm vs. 1.9 vs. 3.1 mm; *p* < 0.01). However, no significant differences were observed for non-painful stimuli between mental simulation and passive observation conditions (Figure 2B). No significant differences were observed between mental simulation and passive observation under the “no visual scene” condition (Figure 2C).

During the passive observation condition, no differences were reported for COP-AP between painful and non-painful situations (Figure 3A). On the other hand, when participants were instructed to imagine themselves in the situation (“mental simulation condition”), COP-AP was displaced posteriorly in the painful situation compared to the non-painful situation at the 4th (−2.1 ± 6.8 mm vs. 1.3 ± 6.5 mm; *p* < 0.05), 11th (−1.2 ± 5.7 mm vs. 1.8 ± 8.0 mm; *p* < 0.05), and 12th seconds (−2.1 ± 6.1 mm vs. 2.1 vs. 9.1 mm; *p* < 0.01; Figure 3B). In Supplementary Table 1, we provide *F*-values and *p*-values reported for COP position in the anteroposterior direction (COP_AP_), heart rate (HR), and electrodermal activity (EDA).

### Physiological responses to visual stimulation

**Figure 4:**
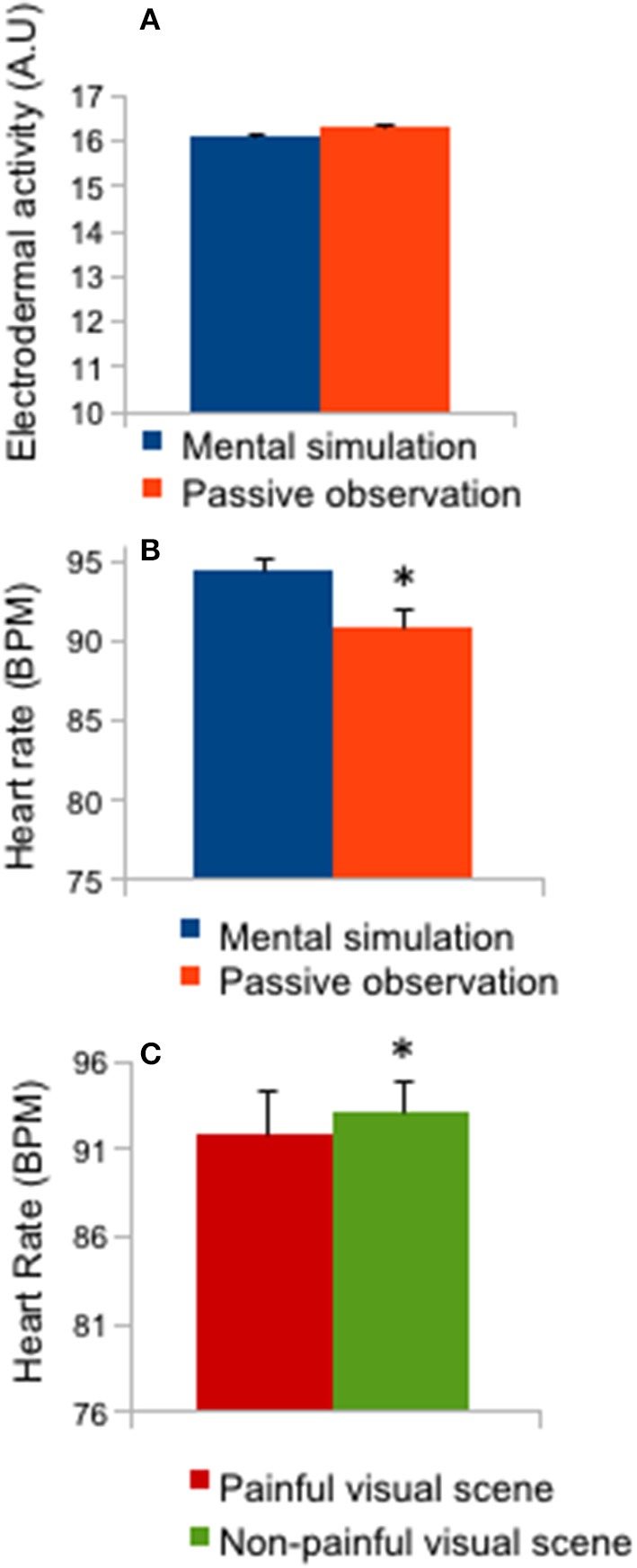
Heart rate and electrodermal activity. **(A)** Mean ± SD of electrodermal activity (mental simulation vs. passive observation conditions); **(B)** Mean ± SD of heart rate (mental simulation vs. passive observation conditions); **(C)** Mean ± SD of heart rate (painful vs. non-painful visual scenes), ^*^p < 0.05.

Figure 4 represents the means and 1-s sliding window of physiological responses during the 12-s presentation of pain or no-pain stimuli. No “time” interaction effects were reported for these physiological data. An effect of instruction (mental simulation/passive observation: *F* = 5.63; *p* < 0.05; effect size: 0.16) was reported for HR (Figure 4B; *p* < 0.05), as the *post-hoc* test revealed a higher HR in the mental simulation condition compared to the passive observation condition (*p* < 0.05). A stimulus effect (*F* = 5.81; *p* < 0.01; effect size = 0.162) was also reported for HR (Figure 4C; *p* < 0.05) as mean HR was lower for painful stimuli than for non-painful stimuli.

## Discussion

This study investigated postural changes and physiological correlates induced by mental simulation of emotional (painful and non-painful) situations. We hypothesized that embodiment of a painful situation—triggered by the instruction to imagine oneself in the situation—would induce a motor response reflected by posterior displacement of the COP-AP position while passive observation of the same visual stimuli would induce lower effects on the mean COP position. Temporal analysis of postural responses was performed while subjects were instructed to either (i) passively observe (no instruction) or (ii) imagine themselves (with instruction) in the depicted painful and non-painful situations. The present study confirms that presentation of emotional stimuli induced bodily responses consisting of postural and physiological changes.

Physiological measurements confirmed a differential reaction of the central nervous system to the emotional content of the stimuli (Lang and Bradley, 2010; Lelard et al., 2014), as HR was significantly lower in the painful condition compared to the non-painful condition. A similar response (lower HR) to an aversive stimulus has been previously described as the expression of bradycardia controlled by the autonomic nervous system (Sgoifo et al., 1999; Baldaro et al., 2001). However, we did not find any differences on temporal analysis for these physiological responses, although we expected an effect on HR (decreased HR in response to aversive stimuli; as reported by Lelard et al., 2014), but not on EDA (depicting slow variations in response to emotional material). The absence of significant variation might be due to a lower degree of arousal with this type of situation (eg. being hit by a hammer on a foot) as compared to those generally presented in previous studies (mutilation scenes). However, HR was significantly higher in the mental simulation condition compared to the passive observation condition, demonstrating the effect of instruction and mental simulation on this physiological response and confirming the efficacy of mental simulation. Horslen and Carpenter (2011) demonstrated that EDA increases with arousal induced by visual stimuli and changes in HR (bradycardia) associated with freezing responses have been previously described (Azevedo et al., 2005; Facchinetti et al., 2006; Stins and Beek, 2007). In the present study, the physiological responses to painful situation were modulated by the instruction given to the subjects to imagine themselves in the depicted situation.

In the present study, we hypothesized a postural difference according to the instructions given to the participants when presented with emotionally charged images (passive observation vs. mental simulation). Our results demonstrate posterior displacement of the COP in the mental simulation condition compared to the passive observation condition. This significant posterior displacement of the COP can be considered to be a defensive response, as previously reported for negative visual stimuli (Hillman et al., 2004; Azevedo et al., 2005; Facchinetti et al., 2006; Stins and Beek, 2007; Lelard et al., 2013, 2014). These results are also in accordance with those of our previous study (Lelard et al., 2013), which demonstrated a freezing-type behavior interpreted as a defensive response during simulation of a painful situation.

The results of this study support the hypothesis that instruction to imagine ourselves in a painful situation activates internal models that lead to an embodiment of the situation (Zahavi, 2008), as modulation of the postural response induced by embodiment of the situation is similar to the modulation reported in previous studies in response to highly disturbing images (mutilation scenes) compared to neutral images (Lelard et al., 2014). When instructed to imagine themselves in the painful situation, the participants adopted a significant posterior displacement of the COP at the 4th second. A similar temporal response to affective images were also reported as participant were watching to aversive images after 3 s (Lelard et al., 2014) or an unpleasant film after 2 s (Hagenaars et al., 2014b). This behavior may reflect a self-protective strategy, inducing threat avoidance and withdrawal behavior in order to protect ourselves (Yamada and Decety, 2009).

A limitation of this study is that we did not counterbalance the passive observation condition and the mental simulation condition, as passive observation always preceded the mental simulation condition. We considered that instructing the participants to imagine themselves in the situation during the first session could have influenced their response during the second session of passive observation. The statistical results of this study must also be interpreted cautiously. The relatively small numbers and significant standard deviations reported in this study highlight the need for further studies to elucidate the mechanisms involved in defensive responses (which may differ from one individual to another depending on each individual's experience).

Embodied cognition has already been demonstrated using motor situations, as postural responses have been previously reported when individuals mentally simulate movement (Rodrigues et al., 2010; Grangeon et al., 2011; Stins et al., 2015). To our knowledge, our study is the first to describe adjustments of postural control in response to mental simulation of affective/motor pictures.

## Author contributions

TL conducted the acquisitions, the statistical analyses and wrote the paper. PK, SA, and OG conducted the statistical analyses and wrote the ethical agreement and funded the research. HM conducted the acquisitions, and wrote the paper.

### Conflict of interest statement

The authors declare that the research was conducted in the absence of any commercial or financial relationships that could be construed as a potential conflict of interest.

## Supplementary material

Supplementary Table 1 *F*-values and *p*-values reported for COP position in the anteroposterior direction (COP_AP_), heart rate (HR), and electrodermal activity (EDA).
