# Supplementary material for: Mental Simulation of Painful Situations Has an Impact on Posture and Psychophysiological Parameters
Source: Front Psychol. 2017 Nov 21;8:2012. doi: 10.3389/fpsyg.2017.02012 (PMC5702461; doi:10.3389/fpsyg.2017.02012)
Supplement: Supplementary Table 1 — F-values and p-values reported for COP position in the anteroposterior direction (COPAP), heart rate (HR), and electrodermal activity (EDA). [file Table1.DOCX]

|  | F value | | | | |  |
| --- | --- | --- | --- | --- | --- | --- |
|  | COPAP |  | HR |  | EDA | |
| instruction | 0.16 |  | 5.63 | p<0.05 | 0.12 | |
| stimuli | 0.51 |  | 5.81 | p<0.01 | 0.67 | |
| time | 1.51 |  | 3.09 |  | 1.89 | |
| instruction x stimuli | 1.77 |  | 2.74 |  | 1.16 | |
| instruction x time | 0.79 |  | 2.37 |  | 1.28 | |
| stimuli x time | 1.54 |  | 0.86 |  | 0.84 | |
| instruction x stimuli x time | 2.01 | p<0.05 | 0.86 |  | 0.73 | |
